# Effectiveness of Pfizer-BioNTech and Moderna Vaccines in Preventing SARS-CoV-2 Infection Among Nursing Home Residents Before and During Widespread Circulation of the SARS-CoV-2 B.1.617.2 (Delta) Variant — National Healthcare Safety Network, March 1–August 1, 2021

**DOI:** 10.15585/mmwr.mm7034e3

**Published:** 2021-08-27

**Authors:** Srinivas Nanduri, Tamara Pilishvili, Gordana Derado, Minn Minn Soe, Philip Dollard, Hsiu Wu, Qunna Li, Suparna Bagchi, Heather Dubendris, Ruth Link-Gelles, John A. Jernigan, Daniel Budnitz, Jeneita Bell, Andrea Benin, Nong Shang, Jonathan R. Edwards, Jennifer R. Verani, Stephanie J. Schrag

**Affiliations:** ^1^CDC COVID-19 Response Team; ^2^Lantana Consulting Group, East Thetford, Vermont.

Nursing home and long-term care facility residents live in congregate settings and are often elderly and frail, putting them at high risk for infection with SARS-CoV-2, the virus that causes COVID-19, and severe COVID-19–associated outcomes; therefore, this population was prioritized for early vaccination in the United States (*1*). Following rapid distribution and administration of the mRNA COVID-19 vaccines (Pfizer-BioNTech and Moderna) under an Emergency Use Authorization by the Food and Drug Administration (*2*), observational studies among nursing home residents demonstrated vaccine effectiveness (VE) ranging from 53% to 92% against SARS-CoV-2 infection (*3*–*6*). However, concerns about the potential for waning vaccine-induced immunity and the recent emergence of the highly transmissible SARS-CoV-2 B.1.617.2 (Delta) variant^†^ highlight the need to continue to monitor VE (*7*). Weekly data reported by the Centers for Medicaid & Medicare (CMS)–certified skilled nursing facilities or nursing homes to CDC’s National Healthcare Safety Network (NHSN)^§^ were analyzed to evaluate effectiveness of full vaccination (2 doses received ≥14 days earlier) with any of the two currently authorized mRNA COVID-19 vaccines during the period soon after vaccine introduction and before the Delta variant was circulating (pre-Delta [March 1–May 9, 2021]), and when the Delta variant predominated^¶^ (Delta [June 21–August 1, 2021]). Using 17,407 weekly reports from 3,862 facilities from the pre-Delta period, adjusted effectiveness against infection for any mRNA vaccine was 74.7% (95% confidence interval [CI] = 70.0%–78.8%). Analysis using 33,160 weekly reports from 11,581 facilities during an intermediate period (May 10–June 20) found that the adjusted effectiveness was 67.5% (95% CI = 60.1%–73.5%). Analysis using 85,593 weekly reports from 14,917 facilities during the Delta period found that the adjusted effectiveness was 53.1% (95% CI = 49.1%–56.7%). Effectiveness estimates were similar for Pfizer-BioNTech and Moderna vaccines. These findings indicate that mRNA vaccines provide protection against SARS-CoV-2 infection among nursing home residents; however, VE was lower after the Delta variant became the predominant circulating strain in the United States. This analysis assessed VE against any infection, without being able to distinguish between asymptomatic and symptomatic presentations. Additional evaluations are needed to understand protection against severe disease in nursing home residents over time. Because nursing home residents might remain at some risk for SARS-CoV-2 infection despite vaccination, multiple COVID-19 prevention strategies, including infection control, testing, and vaccination of nursing home staff members, residents, and visitors, are critical. An additional dose of COVID-19 vaccine might be considered for nursing home and long-term care facility residents to optimize a protective immune response.

Effectiveness of mRNA COVID-19 vaccines against laboratory-confirmed SARS-CoV-2 infection among nursing home residents was evaluated using data reported to NHSN. CMS-certified nursing homes are required to report aggregate weekly numbers of new laboratory-confirmed SARS-CoV-2 infections among residents, by vaccination status (product and number of doses received), to NHSN. Vaccination status of cases was categorized as 1) unvaccinated (no COVID-19 vaccine doses); 2) fully vaccinated with an mRNA vaccine (2 doses ≥14 days before collection of a SARS-CoV-2–positive specimen), and 3) “other” (single dose of mRNA or Janssen [Johnson & Johnson] vaccine or received unspecified vaccines). Nursing homes also reported weekly on the number of residents by vaccination status; reporting on resident vaccination status was voluntary during the pre-Delta period but was required by CMS starting on June 6, 2021.

Facility-level weekly records for the analysis combined case counts by vaccination status in each week with the weekly number of residents by vaccination status 2 weeks previously. This ensured that residents were counted as fully vaccinated only after ≥14 days from receipt of a second dose. Weekly reports of case counts were excluded if a facility did not report resident counts by vaccination status for the corresponding week 2 weeks earlier. Records from facilities with case data during March 1–August 1, 2021, and the corresponding data on resident vaccination status during February 15–July 18, 2021, were combined for an overall 22-week study period. During the study period, 15,254 facilities sent 330,864 weekly reports with case counts to NHSN; of these, 15,236 facilities (99.9%) sent 144,334 (43.6%) weekly reports with counts of residents by vaccination status.

A generalized linear mixed effects model was used with a zero-inflated Poisson distribution (used to model data that have an excess of zero counts) for case counts by vaccination status, offset by resident counts, to estimate the ratio of infection rates among fully vaccinated and unvaccinated residents. To account for variability across sites, facility was included as a random effect. Because of potential for confounding by time, calendar week was modeled as a fixed effect covariate. Nonlinearity of infection rates over calendar weeks was modeled with cubic splines. To evaluate the effect of circulating SARS-CoV-2 variants on VE, the study period was stratified into three periods: 1) pre-Delta (March 1–May 9); 2) intermediate, the period when Delta circulation was documented but not predominant (May 10–June 20); and 3) Delta, when ≥50% of SARS-CoV-2 viruses sequenced were the Delta variant (June 21–August 1), with an interaction term between this categorical time variable and vaccination status to obtain VE estimates for each period. The following characteristics were evaluated as potential confounders of VE: 1) facility-level cumulative SARS-CoV-2 infection rates combined for staff members and residents from May 8, 2020, through the week of reporting; 2) weekly local county incidence of SARS-CoV-2 infections; and 3) CDC Social Vulnerability Index score** for each facility’s county. The change-in-estimate criterion for the regression coefficient with a 10% cutoff was used to evaluate covariates; none met this criterion. VE was estimated as 1 minus the rate ratio multiplied by 100, adjusted for calendar week and facility as a random effect. VE for the “other” category is not presented because this group combines different categories, and estimates would not be meaningful. Data analysis was conducted using SAS (version 9.4; SAS Institute) and R (version 4.0.4; R Foundation); statistical significance was defined as p<0.05. This activity was reviewed by CDC and was conducted consistent with federal laws and institutional policies.^††^

After applying exclusion criteria and combining facility-level weekly case and corresponding resident counts, the analysis included 136,160 reports from 14,997 facilities (median of eight reports per facility; interquartile range = 6–10), with 3,862 (25.8%) facilities reporting during the pre-Delta period, 11,581 (77.2%) during the intermediate period, and 14,917 (99.5%) during the Delta period. Overall, the analysis included 10,428,783 aggregate weekly resident counts, including 1,531,446 (14.7%) unvaccinated residents, 5,174,098 (49.6%) fully vaccinated with Pfizer-BioNTech, 2,633,700 (25.3%) fully vaccinated with Moderna, and 1,089,539 (10.4%) with “other” vaccination status. Overall, 6,879 COVID-19 cases were identified, including 2,113 (30.7%) in unvaccinated residents, 2,603 (37.8%) in residents fully vaccinated with Pfizer-BioNTech, 1,302 (18.9%) in residents fully vaccinated with Moderna, and 861 (12.5%) in residents with “other” vaccination status.

During the pre-Delta period, adjusted VE against infection among those fully vaccinated (versus unvaccinated) was 74.7% for any mRNA vaccine, 74.2% for Pfizer-BioNTech, and 74.7% for Moderna (Table). During the Delta period, adjusted VE against infection among those fully vaccinated was 53.1% for any mRNA vaccine, 52.4% for Pfizer-BioNTech, and 50.6% for Moderna. VE estimates for the Delta period were significantly lower than those for the pre-Delta period (p<0.001). VE point estimates during the intermediate period were lower than those during the pre-Delta period; however, the estimates were not significantly different (p = 0.06) (Table).

**TABLE:** Effectiveness of full vaccination* with Pfizer-BioNTech or Moderna vaccines in preventing SARS-CoV-2 infection among nursing home residents, by period of B.1.617.2 (Delta) variant circulation — National Healthcare Safety Network, March 1–August 1, 2021

Vaccine type/Period^†^	Aggregate weekly count of residents	No. of cases	Vaccine effectiveness, % (95% CI)		p-value**
			Unadjusted^§^	Adjusted^¶^	
**Any mRNA vaccine**					
Period 1: pre-Delta	936,123	466	74.3 (69.5–78.4)	74.7 (70.0–78.8)	Ref
Period 2: intermediate	1,859,929	440	65.8 (58.5–71.9)	67.5 (60.1–73.5)	0.06
Period 3: Delta	5,011,746	2,999	52.8 (48.8–56.5)	53.1 (49.1–56.7)	<0.001
**Pfizer-BioNTech**					
Period 1: pre-Delta	679,288	348	74.7 (69.5–79.0)	74.2 (68.9–78.7)	Ref
Period 2: intermediate	1,246,078	316	63.5 (54.9–70.5)	66.5 (58.3–73.1)	0.07
Period 3: Delta	3,248,732	1,939	52.2 (47.7–56.3)	52.4 (48.0–56.4)	<0.001
**Moderna**					
Period 1: pre-Delta	256,835	118	72.6 (66.1–77.8)	74.7 (66.2–81.1)	Ref,
Period 2: intermediate	613,851	124	73.2 (66.8–78.3)	70.4 (60.1–78.0)	0.45
Period 3: Delta	1,763,014	1,060	48.4 (42.3–53.8)	50.6 (45.0–55.7)	<0.001
**Unvaccinated**					
Period 1: pre-Delta	217,534	447	Ref		NA
Period 2: intermediate	360,051	269			
Period 3: Delta	953,861	1,397			

**Abbreviations:** CI = confidence interval; NA = not applicable; Ref = referent group.

* Fully vaccinated cases were defined as infections in residents who received the second of 2 doses of either Pfizer-BioNTech or Moderna vaccines ≥14 days before SARS-CoV-2–positive specimen collection.

^†^ Periods for analysis were stratified as follows: period 1 = pre-Delta (March 1–May 9, 2021); period 2 = intermediate (May 10–June 20, 2021); period 3 = Delta (June 21–August 1, 2021).

^§^ Results from a generalized linear mixed effects model with random effects for facility and zero-inflated Poisson distribution; vaccine effectiveness was estimated as 1 minus the rate ratio multiplied by 100, with rate ratio comparing rates among fully vaccinated to those among unvaccinated persons. Results for “other” category, which included those who received a single dose of Janssen (Johnson & Johnson) or mRNA vaccine, or those residents who received unspecified vaccines are not presented because this group combines the different categories and estimates will not be meaningful.

**^¶^** Results from the same model controlling for calendar week of reporting of case counts.

** p-values for comparison of adjusted vaccine effectiveness estimates in period 2 and period 3 with estimates in period 1. The difference in estimates among periods was evaluated by adding an interaction between periods and vaccine status in the model.

## Discussion

Analysis of nursing home COVID-19 data from NHSN indicated a significant decline in effectiveness of full mRNA COVID-19 vaccination against laboratory-confirmed SARS-CoV-2 infection, from 74.7% during the pre-Delta period (March 1–May 9, 2021) to 53.1% during the period when the Delta variant predominated in the United States. This study could not differentiate the independent impact of the Delta variant from other factors, such as potential waning of vaccine-induced immunity. Further research on the possible impact of both factors on VE among nursing home residents is warranted. Because nursing home residents might remain at some risk for SARS-CoV-2 infection despite vaccination, multipronged COVID-19 prevention strategies, including infection control,^§§^ testing, and vaccination of nursing home staff members, residents, and visitors are critical.

These results (pre-Delta 74.7%; Delta 53.1%) fall within the range of findings from other studies of COVID-19 mRNA VE in nursing home residents conducted before the Delta variant was prevalent, with estimates against infection ranging from 53% to 92% (*3*–*6*). Variability in VE estimates across studies can result from differences in underlying populations, SARS-CoV-2 testing practices and diagnostics, prevalence of previous infections, analytic methods, and virus variant strains in circulation.

Nursing home residents, who are often elderly and frail, might have a less robust response to vaccines, and are at higher risk for infection with SARS-CoV-2 and for severe COVID-19 (*8*). In addition, nursing home residents were among the earliest groups vaccinated in the United States; thus, if vaccine-induced immunity does wane over time, this decrease in VE might first be observed among nursing home residents. Because increased U.S. circulation of the Delta variant coincided with a period ≥6 months after vaccine introduction, the extent to which reduced vaccine protection against Delta and potential waning immunity contributed to the lower VE in the Delta period could not be determined by this study.

Nursing homes were aggressive in case ascertainment because of guidelines recommending weekly point prevalence surveys if a single SARS-CoV-2 infection in a staff member or resident was identified.^¶¶^ This analysis assessed VE against any infection, without being able to distinguish between asymptomatic and symptomatic infections. Additional evaluations are needed to understand protection against severe disease in nursing home residents over time.

The findings in this report are subject to at least five limitations. First, resident-level demographic or clinical data were not reported to NHSN. Therefore, the analysis could not control for potential confounders, such as age, presence of underlying health conditions, or the influence of previous SARS-CoV-2 infections on VE. Second, vaccination dates were not available and time since vaccination could not be measured to evaluate potential waning of protection. Third, staff member vaccination data were not sufficiently complete to assess as a potential confounder. Fourth, before June 7, 2021, weekly reporting of resident vaccination status was voluntary, and missing data limited inclusion of facility records during this period. Although the magnitude of potential bias introduced by missing data could not be assessed, a bias indicator analysis was conducted, which indicated that VE was likely underestimated during the pre-Delta period (COVID-19 Vaccine Effectiveness Team, CDC, unpublished data, 2021). Finally, the study assessed only nursing home residents and is not generalizable to the broader population.

Both Pfizer-BioNTech and Moderna mRNA vaccines were highly effective in preventing SARS-CoV-2 infection in nursing home residents early after vaccine introduction. However, the effectiveness among this population in recent months has been significantly lower. To prevent transmission of SARS-CoV-2 in nursing homes, these findings highlight the critical importance of COVID-19 vaccination of staff members, residents, and visitors and adherence to rigorous COVID-19 prevention strategies. An additional dose of COVID-19 vaccine might be considered for nursing home and long-term care facility residents to optimize a protective immune response.***

**Summary**

**What is already known about this topic?**

Early observational studies among nursing home residents showed mRNA vaccines to be 53% to 92% effective against SARS-CoV-2 infection.

**What is added by this report?**

Two doses of mRNA vaccines were 74.7% effective against infection among nursing home residents early in the vaccination program (March–May 2021). During June–July 2021, when B.1.617.2 (Delta) variant circulation predominated, effectiveness declined significantly to 53.1%.

**What are the implications for public health practice?**

Multicomponent COVID-19 prevention strategies, including vaccination of nursing home staff members, residents, and visitors, are critical. An additional dose of COVID-19 vaccine might be considered for nursing home and long-term care facility residents to optimize a protective immune response.

## Footnotes

* These authors contributed equally to this report.

†
https://www.cdc.gov/coronavirus/2019-ncov/variants/delta-variant.html

§
https://www.cdc.gov/nhsn/ltc/covid19/index.html

¶
https://covid.cdc.gov/covid-data-tracker/#variant-proportions

**
https://www.atsdr.cdc.gov/placeandhealth/svi/data_documentation_download.html

†† 45 C.F.R. part 46, 21 C.F.R. part 56; 42 U.S.C. Sect. 241(d); 5 U.S.C. Sect. 552a; 44 U.S.C. Sect. 3501 et seq.

§§
https://www.cdc.gov/coronavirus/2019-ncov/hcp/long-term-care.html

¶¶
https://www.cms.gov/files/document/qso-20-38-nh.pdf

***
https://www.cdc.gov/vaccines/acip/meetings/slides-2021-08-13.html

## References

[1] Dooling K, Marin M, Wallace M, The Advisory Committee on Immunization Practices’ updated interim recommendation for allocation of COVID-19 vaccine—United States, December 2020. MMWR Morb Mortal Wkly Rep 2021;69:1657–60. 10.15585/mmwr.mm695152e2 33382671 PMC9191902

[2] Gharpure R, Patel A, Link-Gelles R. First-dose COVID-19 vaccination coverage among skilled nursing facility residents and staff. JAMA 2021;325:1670–1. 10.1001/jama.2021.2352 33625464 PMC8722356

[3] Monge S, Olmedo C, Alejos B, Lapeña MF, Sierra MJ, Limia A. Direct and indirect effectiveness of mRNA vaccination against SARS-CoV-2 infection in long-term care facilities in Spain. Emerg Infect Dis 2021. Epub July 27, 2021. 10.3201/eid2710.211184 PMC8462307 34314670

[4] Mazagatos C, Monge S, Olmedo C, ; Working Group for the surveillance and control of COVID-19 in Spain. Effectiveness of mRNA COVID-19 vaccines in preventing SARS-CoV-2 infections and COVID-19 hospitalisations and deaths in elderly long-term care facility residents, Spain, weeks 53 2020 to 13 2021. Euro Surveill 2021;26:2100452. 10.2807/1560-7917.ES.2021.26.24.2100452 34142647 PMC8212595

[5] Britton A, Jacobs Slifka KM, Edens C, Effectiveness of the Pfizer-BioNTech COVID-19 vaccine among residents of two skilled nursing facilities experiencing COVID-19 outbreaks—Connecticut, December 2020–February 2021. MMWR Morb Mortal Wkly Rep 2021;70:396–401. 10.15585/mmwr.mm7011e3 33735160 PMC7976620

[6] Emborg H-D, Valentiner-Branth P, Schelde AB, Vaccine effectiveness of the BNT162b2 mRNA COVID-19 vaccine against RT-PCR confirmed SARS-CoV-2 infections, hospitalisations and mortality in prioritised risk groups. medRxiv [Preprint posted online June 2, 2021]. https://www.medrxiv.org/content/10.1101/2021.05.27.21257583v1

[7] Puranik A, Lenehan PJ, Silvert E, Comparison of two highly-effective mRNA vaccines for COVID-19 during periods of Alpha and Delta variant prevalence. medRxiv [Preprint posted online August 9, 2021]. https://www.medrxiv.org/content/10.1101/2021.08.06.21261707v2

[8] Bagchi S, Mak J, Li Q, Rates of COVID-19 among residents and staff members in nursing homes—United States, May 25–November 22, 2020. MMWR Morb Mortal Wkly Rep 2021;70:52–5. 10.15585/mmwr.mm7002e2 33444301 PMC7808710

